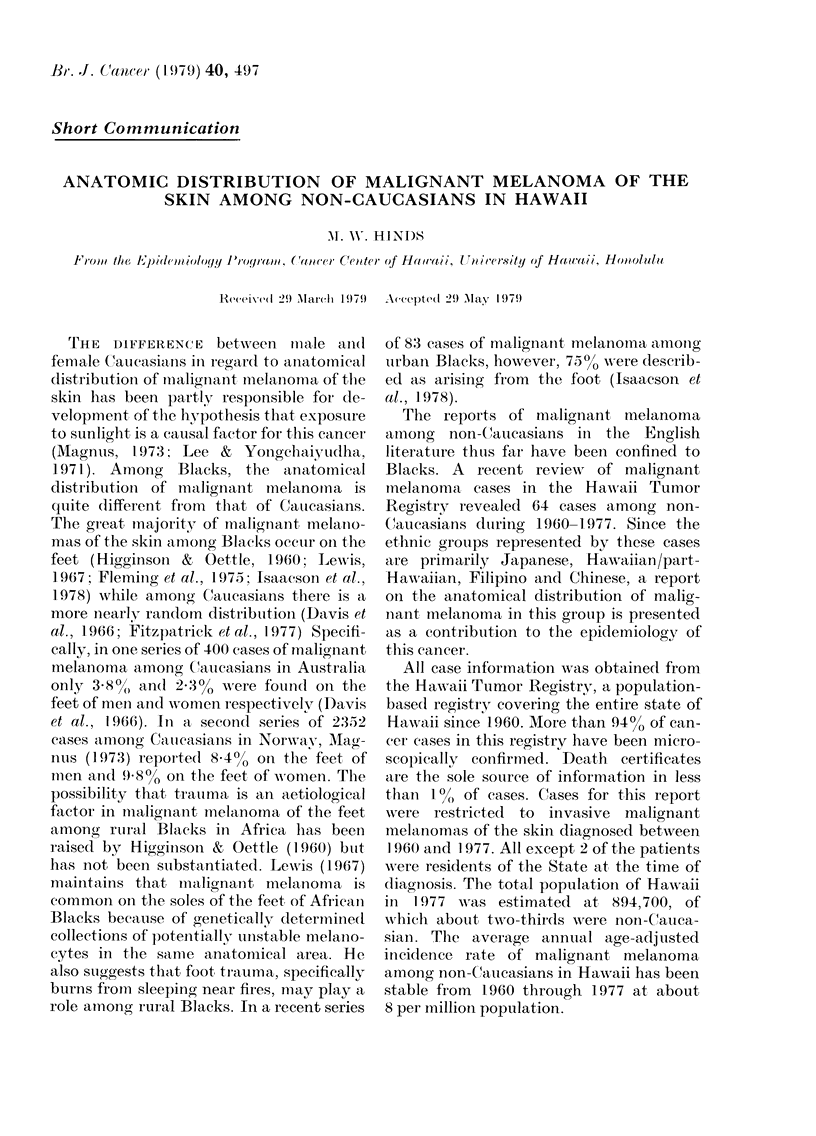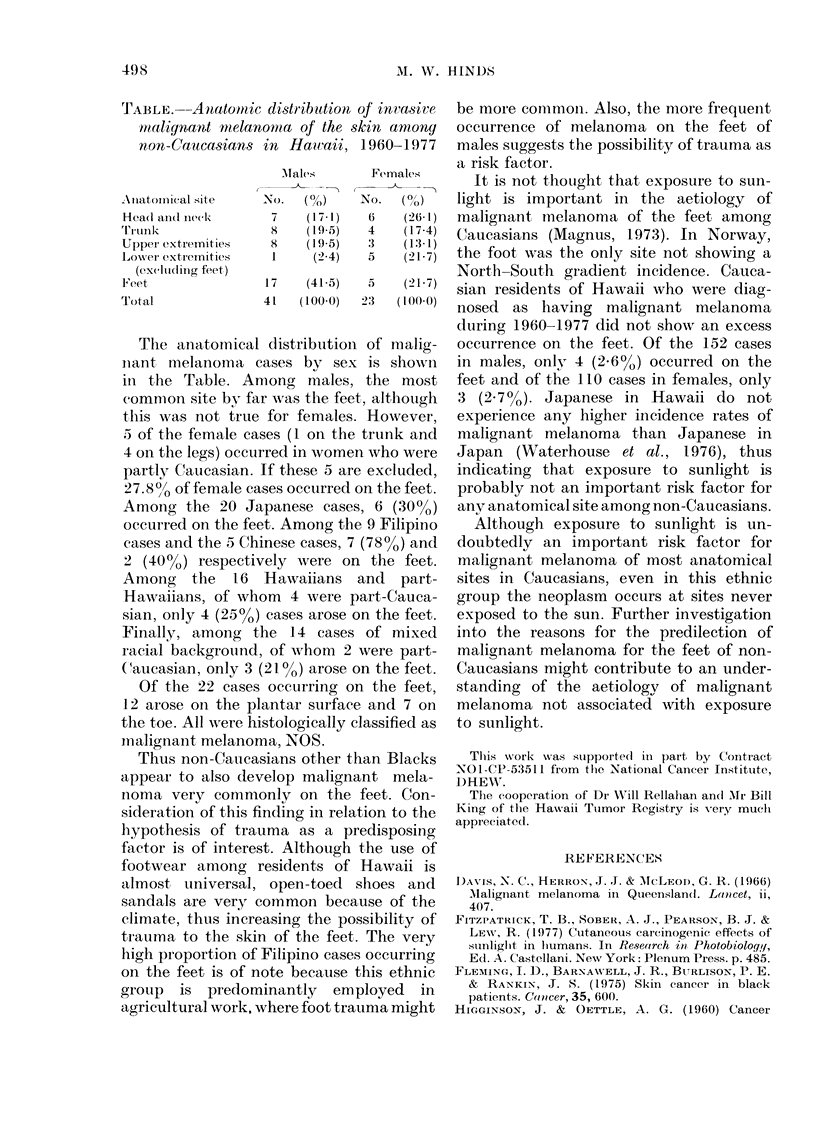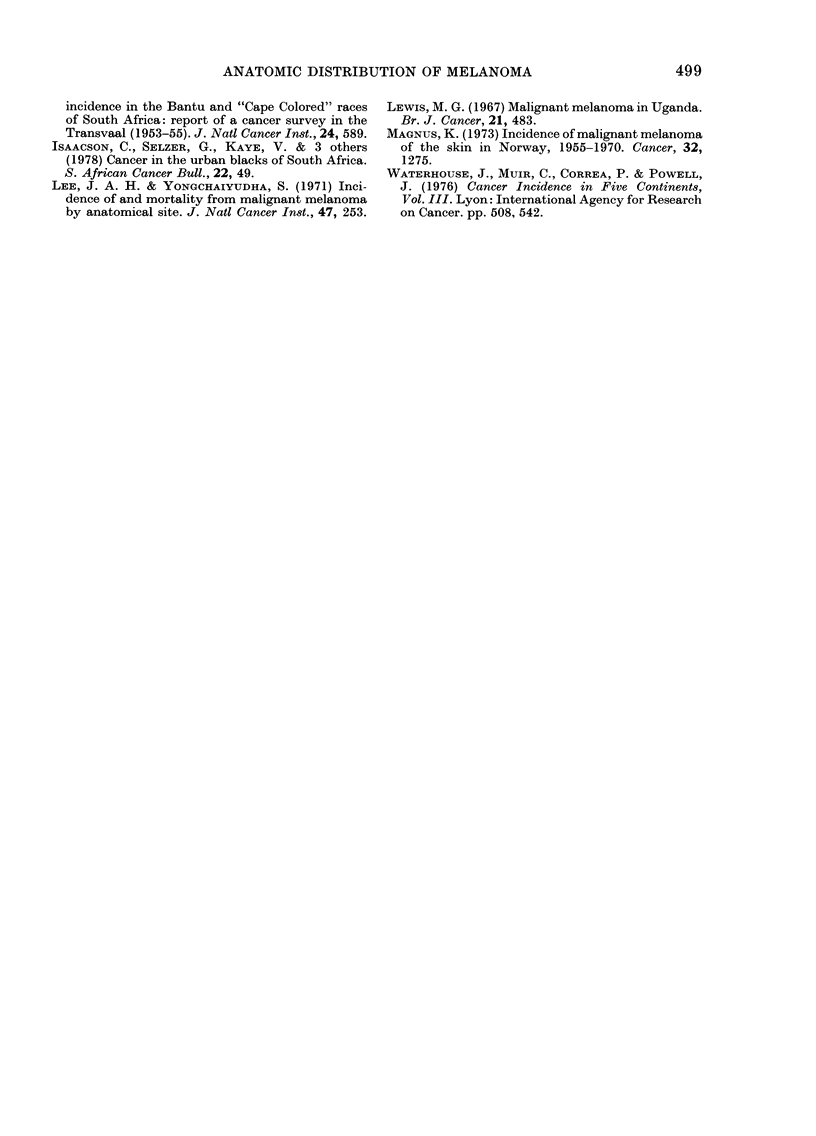# Anatomic distribution of malignant melanoma of the skin among non-Caucasians in Hawaii.

**DOI:** 10.1038/bjc.1979.207

**Published:** 1979-09

**Authors:** M. W. Hinds


*Br. J. Cancer* (1979) **40**, 497

***Short Communication***

### ANATOMIC DISTRIBUTION OF MALIGNANT MELANOMA OF THE SKIN AMONG NON-CAUCASIANS IN HAWAII

M. W. HINDS

*From the Epidemiology Program, Cancer Center of Hawaii, University of Hawaii, Honolulu*

Received 29 March 1979 Accepted 29 May 1979

THE DIFFERENCE between male and female Caucasians in regard to anatomical distribution of malignant melanoma of the skin has been partly responsible for development of the hypothesis that exposure to sunlight is a causal factor for this cancer (Magnus, 1973; Lee & Yongchaiyudha, 1971). Among Blacks, the anatomical distribution of malignant melanoma is quite different from that of Caucasians. The great majority of malignant melanomas of the skin among Blacks occur on the feet (Higginson & Oettle, 1960; Lewis, 1967; Fleming *et al.*, 1975; Isaacson *et al.*, 1978) while among Caucasians there is a more nearly random distribution (Davis *et al.*, 1966; Fitzpatrick *et al.*, 1977) Specifically, in one series of 400 cases of malignant melanoma among Caucasians in Australia only 3·8% and 2·3% were found on the feet of men and women respectively (Davis *et al.*, 1966). In a second series of 2352 cases among Caucasians in Norway, Magnus (1973) reported 8·4% on the feet of men and 9·8% on the feet of women. The possibility that trauma is an aetiological factor in malignant melanoma of the feet among rural Blacks in Africa has been raised by Higginson & Oettle (1960) but has not been substantiated. Lewis (1967) maintains that malignant melanoma is common on the soles of the feet of African Blacks because of genetically determined collections of potentially unstable melanocytes in the same anatomical area. He also suggests that foot trauma, specifically burns from sleeping near fires, may play a role among rural Blacks. In a recent series of 83 cases of malignant melanoma among urban Blacks, however, 75% were described as arising from the foot (Isaacson *et al.*, 1978).

The reports of malignant melanoma among non-Caucasians in the English literature thus far have been confined to Blacks. A recent review of malignant melanoma cases in the Hawaii Tumor Registry revealed 64 cases among non-Caucasians during 1960–1977. Since the ethnic groups represented by these cases are primarily Japanese, Hawaiian/part-Hawaiian, Filipino and Chinese, a report on the anatomical distribution of malignant melanoma in this group is presented as a contribution to the epidemiology of this cancer.

All case information was obtained from the Hawaii Tumor Registry, a population-based registry covering the entire state of Hawaii since 1960. More than 94% of cancer cases in this registry have been microscopically confirmed. Death certificates are the sole source of information in less than 1% of cases. Cases for this report were restricted to invasive malignant melanomas of the skin diagnosed between 1960 and 1977. All except 2 of the patients were residents of the State at the time of diagnosis. The total population of Hawaii in 1977 was estimated at 894,700, of which about two-thirds were non-Caucasian. The average annual age-adjusted incidence rate of malignant melanoma among non-Caucasians in Hawaii has been stable from 1960 through 1977 at about 8 per million population.

TABLE.—*Anatomic distribution of invasive malignant melanoma of the skin among non-Caucasians in Hawaii*, 1960–1977

| Anatomical site | Males No. | Males (%) | Females No. | Females (%) |
|---|---|---|---|---|
| Head and neck | 7 | (17·1) | 6 | (26·1) |
| Trunk | 8 | (19·5) | 4 | (17·4) |
| Upper extremities | 8 | (19·5) | 3 | (13·1) |
| Lower extremities (excluding feet) | 1 | (2·4) | 5 | (21·7) |
| Feet | 17 | (41·5) | 5 | (21·7) |
| Total | 41 | (100·0) | 23 | (100·0) |

The anatomical distribution of malignant melanoma cases by sex is shown in the Table. Among males, the most common site by far was the feet, although this was not true for females. However, 5 of the female cases (1 on the trunk and 4 on the legs) occurred in women who were partly Caucasian. If these 5 are excluded, 27.8% of female cases occurred on the feet. Among the 20 Japanese cases, 6 (30%) occurred on the feet. Among the 9 Filipino cases and the 5 Chinese cases, 7 (78%) and 2 (40%) respectively were on the feet. Among the 16 Hawaiians and part-Hawaiians, of whom 4 were part-Caucasian, only 4 (25%) cases arose on the feet. Finally, among the 14 cases of mixed racial background, of whom 2 were part-Caucasian, only 3 (21%) arose on the feet.

Of the 22 cases occurring on the feet, 12 arose on the plantar surface and 7 on the toe. All were histologically classified as malignant melanoma, NOS.

Thus non-Caucasians other than Blacks appear to also develop malignant melanoma very commonly on the feet. Consideration of this finding in relation to the hypothesis of trauma as a predisposing factor is of interest. Although the use of footwear among residents of Hawaii is almost universal, open-toed shoes and sandals are very common because of the climate, thus increasing the possibility of trauma to the skin of the feet. The very high proportion of Filipino cases occurring on the feet is of note because this ethnic group is predominantly employed in agricultural work, where foot trauma might be more common. Also, the more frequent occurrence of melanoma on the feet of males suggests the possibility of trauma as a risk factor.

It is not thought that exposure to sunlight is important in the aetiology of malignant melanoma of the feet among Caucasians (Magnus, 1973). In Norway, the foot was the only site not showing a North–South gradient incidence. Caucasian residents of Hawaii who were diagnosed as having malignant melanoma during 1960–1977 did not show an excess occurrence on the feet. Of the 152 cases in males, only 4 (2·6%) occurred on the feet and of the 110 cases in females, only 3 (2·7%). Japanese in Hawaii do not experience any higher incidence rates of malignant melanoma than Japanese in Japan (Waterhouse *et al.*, 1976), thus indicating that exposure to sunlight is probably not an important risk factor for any anatomical site among non-Caucasians.

Although exposure to sunlight is undoubtedly an important risk factor for malignant melanoma of most anatomical sites in Caucasians, even in this ethnic group the neoplasm occurs at sites never exposed to the sun. Further investigation into the reasons for the predilection of malignant melanoma for the feet of non-Caucasians might contribute to an understanding of the aetiology of malignant melanoma not associated with exposure to sunlight.

This work was supported in part by Contract NO1-CP-53511 from the National Cancer Institute, DHEW.

The cooperation of Dr Will Rellahan and Mr Bill King of the Hawaii Tumor Registry is very much appreciated.

### REFERENCES

DAVIS, N. C., HERRON, J. J. & McLEOD, G. R. (1966) Malignant melanoma in Queensland. *Lancet*, ii, 407.

FITZPATRICK, T. B., SOBER, A. J., PEARSON, B. J. & LEW, R. (1977) Cutaneous carcinogenic effects of sunlight in humans. In *Research in Photobiology*, Ed. A. Castellani. New York: Plenum Press. p. 485.

FLEMING, I. D., BARNAWELL, J. R., BURLISON, P. E. & RANKIN, J. S. (1975) Skin cancer in black patients. *Cancer*, **35**, 600.

HIGGINSON, J. & OETTLE, A. G. (1960) Cancer incidence in the Bantu and "Cape Colored" races of South Africa: report of a cancer survey in the Transvaal (1953–55). *J. Natl Cancer Inst.*, **24**, 589.

ISAACSON, C., SELZER, G., KAYE, V. & 3 others (1978) Cancer in the urban blacks of South Africa. *S. African Cancer Bull.*, **22**, 49.

LEE, J. A. H. & YONGCHAIYUDHA, S. (1971) Incidence of and mortality from malignant melanoma by anatomical site. *J. Natl Cancer Inst.*, **47**, 253.

LEWIS, M. G. (1967) Malignant melanoma in Uganda. *Br. J. Cancer*, **21**, 483.

MAGNUS, K. (1973) Incidence of malignant melanoma of the skin in Norway, 1955–1970. *Cancer*, **32**, 1275.

WATERHOUSE, J., MUIR, C., CORREA, P. & POWELL, J. (1976) *Cancer Incidence in Five Continents, Vol. III.* Lyon: International Agency for Research on Cancer. pp. 508, 542.